# Editorial: Integrated strategies for lifelong health: multidimensional approaches to aging and lifestyle interventions

**DOI:** 10.3389/fpubh.2026.1777378

**Published:** 2026-01-19

**Authors:** Mingyue Shi, Wei Ge

**Affiliations:** Department of General Practice, Xijing Hospital, Fourth Military Medical University, Xi'an, Shaanxi, China

**Keywords:** aging, integrated strategies, lifelong health, lifestyle interventions, multidimensional approaches

The irreversible global trend of population aging is reshaping our social fabric with unprecedented force and posing severe challenges to public health systems. Traditional research on healthy aging has often focused on single diseases or isolated physiological systems. However, aging is inherently a complex, multidimensional, and dynamic lifelong process, intricately woven from the interplay of biological, psychological, social, behavioral, and environmental factors. Consequently, while fragmented, issue-specific interventions are necessary, they are insufficient to address the systemic challenges posed by population aging. The Research Topic “*Comprehensive Strategies for Lifelong Health: A Multidimensional Aging Approach and Lifestyle Interventions*” emerges precisely against this backdrop. This Research Topic aims to transcend traditional paradigms by bringing together cutting-edge, multidisciplinary research from around the globe to collectively demonstrate and construct an integrated framework for healthy aging. Its core objective is to elucidate that only by integrating multidimensional assessment methods, a life-course prevention philosophy, and coordinated multi-domain lifestyle interventions can we systematically enhance population health reserves and ultimately achieve the overarching goal of extending “healthspan” rather than merely “lifespan.” The articles included in this Research Topic unanimously emphasize that healthy aging is not an exclusive concern of the older adults but a lifelong endeavor commencing in young and middle adulthood, whose intervention strategies must be proactive, systematic, and highly individualized.

## 1 Precision risk assessment and early warning: the cornerstone of comprehensive strategies

Effective intervention begins with precise identification. Multiple studies in this Research Topic demonstrate how modern technologies are empowering early risk assessment in aging health. For instance, research such as “*Development and validation of an explainable machine learning model for predicting the risk of sleep disorders in older adults with multimorbidity: a cross-sectional study*” (Wang X. et al.) utilizes interpretable machine learning models, including Gradient Boosting Machine (GBM), to accurately identify key predictors of sleep disorders such as frailty, cognitive status, nutritional status, living alone, and depression. This approach not only enables early risk quantification but also reveals interactions among factors through methods like SHAP values, providing clinicians with clear operational insights. Similarly, the study “*Socioeconomic status and lifestyle as factors of multimorbidity among older adults in China: Results from the China Health and Retirement Longitudinal Survey*” (Gong et al.) found that, compared to traditional linear models, machine learning algorithms like XGBoost more precisely capture complex risk patterns for multimorbidity and reveal that educational level, functional status (ADL), and socioeconomic factors are more central drivers than individual lifestyle elements. Collectively, these studies emphasize that precision risk assessment based on big data and artificial intelligence forms a solid foundation for implementing targeted comprehensive interventions, facilitating a shift in public health and clinical practice from “reactive treatment” to “proactive prevention.”

## 2 The pivotal role of lifestyle interventions: from evidence to practice

Lifestyle serves as the central theme permeating this Research Topic, with substantial evidence confirming its core value in maintaining multidimensional health in older adults. Physical activity has been consistently demonstrated as fundamental to preserving functional capacity and intrinsic capacity. A meta-analysis on “*Effects of Tai Chi and Baduanjin on Muscle Mass, Muscle Function, and Activities of Daily Living in Individuals with Sarcopenia: A Systematic Review and Meta-Analysis*” (Yang et al.) revealed that these mind-body exercises significantly improve grip strength, gait speed, and muscle mass, highlighting integrated benefits combining strength, balance, and psychological regulation. The study “*Optimizing Resistance Training for Pain Management in Knee and Hip Osteoarthritis: A Pairwise and Dose-Response Meta-Analysis*” (Liu W. et al.) further refined this approach, identifying the optimal weekly training dosage (680 MET/min/week) for pain relief through dose-response analysis and noting that effects are moderated by age and sex—emphasizing the importance of individualized prescription. Broader perspectives examine specific activity modalities: studies such as “*Feasibility and effects of horticultural activities on frailty, physical function, and quality of life among older adult residents in nursing homes: A quasi-experimental study*” (Wang J. et al.) and “*Effects of Agricultural or Gardening Physical Activity on Cardiovascular Disease and Dementia-Related Markers via Arterial Stiffness, Cognitive Function, and Cerebral White Matter Status: Results from Cross-sectional and Interventional Studies*” (Nishiwaki et al.) found that such activities not only enhance physical function but may also confer dual cardiocerebral benefits by improving arterial stiffness, cognitive function, and white matter health, simultaneously engaging physical, cognitive, and social domains. Nutrition constitutes another critical pillar. The “*The Mushrooms on the Menu (MOM) Study: Vitamin D mushrooms (UV-exposed) are a feasible and acceptable way to increase vitamin D intake in a residential aged care facility*” (Ferraris et al.) offers an exemplary “food-first” approach, demonstrating that incorporating UVB-treated mushrooms into aged care meals is a feasible, acceptable, and effective strategy to significantly increase vitamin D intake, providing innovative insights for preventing malnutrition. Multiple studies (e.g., sleep disorder prediction, cognitive impairment association analyses) consistently identify nutritional status as a key determinant of health outcomes, underscoring the necessity of integrating personalized nutritional support into comprehensive strategies. Sleep and cognitive health represent a crucial intersection for integrated interventions. The study “*Associations Between Sleep Duration, Physical Activity, and Cognitive Impairment in Older Adults-Empirical Analysis Based on CHARLS Data*” (Ma et al.) revealed a U-shaped relationship: both insufficient and excessive sleep duration increase cognitive impairment risk, with significant synergistic negative effects when combined with low physical activity levels. This indicates that interventions targeting cognitive health must concurrently address sleep management and physical activity promotion, fostering a virtuous cycle of “sleep-activity-cognition.”

## 3 Psychosocial and environmental support: the ecosystem for healthy aging

Healthy aging is inextricably linked to a supportive psychosocial environment. Multiple articles in this Research Topic profoundly elucidate the pivotal role of social determinants. Social Connection and Mental Health: Studies such as “*The Hidden Network: Community Sense, Social Desirability, and Their Protective Influence on Negative Emotions in Aging Populations*” (Chu et al.) and “*The Impact of Life Events on Health-Related Quality of Life in Rural Older Adults: The Moderating Role of Social Support*” (Liu L. et al.) demonstrate that social capital, social support, and a sense of community can significantly buffer the detrimental health impacts of adverse life events and psychological stress. Going a step further, a network analysis in “*Inter-relationships of depression and anxiety symptoms among widowed and non-widowed older adults: Findings from the Chinese Longitudinal Healthy Longevity Survey (CLHLS) based on network analysis and propensity score matching*” (Li et al.) meticulously delineates unique “bridge symptoms” (e.g., the strong connection between “Feeling lonely” and “Nervous”) within the widowed cohort, providing a precise map for targeted psychological interventions.

The physical environment is equally critical. Research from “*The Association between home Modifications and depression among Older people in China*” (Lyu and Sun) and “*Living arrangements, health outcomes, and the buffering role of social capital among older adults in China*” (Zheng and Ni) confirms that appropriate home modifications and positive living arrangements (e.g., co-residing with family) are significantly associated with reduced depressive symptoms and enhanced self-rated health. Social capital (e.g., frequent visits from children, community engagement) serves as a crucial buffer in this relationship. These findings collectively suggest that building “age-friendly” communities and living environments is an indispensable component in supporting older adults to achieve the goal of “aging in place.”

## 4 From research to practice: systemic pathways for implementing comprehensive strategies

The ultimate objective of this Research Topic lies in translating the multidimensional evidence presented into real-world practice.

Innovative Service Models: The study “*From the WHO Framework to Integrated Senior Health and Wellness Hub Program: An Implementation Journey*” (Huang X. et al.) provides a paradigm for operationalizing the WHO-COPE framework. It demonstrates how to construct an integrated care system within actual community settings through community-driven capacity assessment, nutritional education, exercise programs, and a “train-the-trainer” model, effectively merging health and social services. Similarly, the “*Study protocol for the randomized controlled trial of EMBOLDEN: a multifaceted intervention aimed at Enhancing physical and community Mobility in older adults with health inequities using community co-design*” (Ganann et al.) aims to co-design an intervention integrating physical activity, nutrition, social participation, and system navigation for older adults facing health inequities.

Policy and Technological Enablers: Furthermore, “*The smart senior care demand in China in the context of active ageing: A qualitative study with multiple perspectives*” (Huang Q. et al.) drawing from stakeholder insights, identifies key challenges for smart senior care development—including user acceptance and cost—and emphasizes the need to address these through multi-stakeholder engagement alongside legal and technological safeguards to meet the comprehensive health, daily living, and social care needs of older adults. A pilot study, “*Efficacy of extracting and preventively intervening late-stage older adults who are at high risk for spending high medical costs by using the health check-up system in Japan: A pilot study*” (Kazawa et al.) illustrates how comprehensive risk assessment can be embedded within existing public health systems (e.g., senior health checkups) to deliver targeted early interventions for preventing costly hospitalizations.

## 5 Conclusion and future perspectives

In summary, this Research Topic compellingly demonstrates, through robust evidence from epidemiology, clinical medicine, public health, social sciences, and data science, that the path toward healthy aging is unequivocally one of “comprehensive strategies.” This necessitates: 1. A Shift in Perspective: Transitioning from a focus on single diseases to a holistic concern for an individual's “intrinsic capacity” and “functional ability,” and from reactive treatment to lifelong proactive prevention. 2. Integration of Interventions: Dismantling disciplinary silos to systematically combine interventions—encompassing physical activity, nutrition, cognitive training, psychological support, social engagement, and environmental adaptations—into multi-component intervention packages. 3. Technological Empowerment: Leveraging big data, artificial intelligence, and digital health technologies to enable precise risk prediction, individualized intervention tailoring, and dynamic monitoring of outcomes. 4. Systemic Collaboration: Strengthening synergy among healthcare systems, social services, community environments, and policymakers to collectively construct a supportive ecosystem for lifelong health.

Future research should continue to deepen the exploration of synergistic mechanisms among various interventions, develop more efficient integrated models, and specifically address the implementation challenges in disseminating these strategies among resource-limited settings and vulnerable groups. We firmly believe that steadfast adoption and implementation of this multidimensional, comprehensive approach will not only empower individuals to achieve healthier and more vibrant lives in their later years but also contribute to building a more resilient and inclusive society for all ages.

